# Dynamic control of heat flow using a spin-chain ladder cuprate film and an ionic liquid

**DOI:** 10.1038/s41598-020-70835-z

**Published:** 2020-09-02

**Authors:** Nobuaki Terakado, Yoshinori Nara, Yuki Machida, Yoshihiro Takahashi, Takumi Fujiwara

**Affiliations:** 1 Department of Applied Physics, Tohoku University, 6-6-05 Aoba, Aoba-ku, Sendai, 980-8579 Japan; 2 JST, PRESTO, 4-1-8 Honcho, Kawaguchi, 332-0012 Japan

**Keywords:** Electrical and electronic engineering, Materials science, Applied physics, Condensed-matter physics

## Abstract

Dynamic control of heat flow for applications in thermal management has attracted much interest in fields such as electronics and thermal engineering. Spin-chain ladder cuprates are promising materials to realize dynamic control of heat flow, since their magnon thermal conductivity is sensitive to the hole density in the spin ladders, which can be dynamically controlled by an external field. Here, we demonstrate the electric control of heat flow using a polycrystalline cuprate film and an ionic liquid. The results showed that a voltage application to the interface causes imperfectly recoverable decreases in both the thermal conductance of the film and the peak due to magnons in the Raman spectra. This result may be attributed to an increase in the hole density in the spin ladders. This report highlights that magnon thermal conduction has potential for the development of advanced thermal management applications.

## Introduction

Recently, dynamic control of heat flow, such as via thermal conductivity control using field-effect changes in material properties^1–4^ and the spin Seebeck effect^5,6^, has attracted much interest and has been demonstrated. These control methods have great potential for advanced thermal management, including active heat dissipation, storage, and switching, as they provide stability in highly integrated electronic devices and enable effective reuse of waste heat; moreover, these methods allow temporal-spatial and precise control of temperature in devices and materials whose performance may decrease due to temperature perturbation^3^.

**Figure 1:**
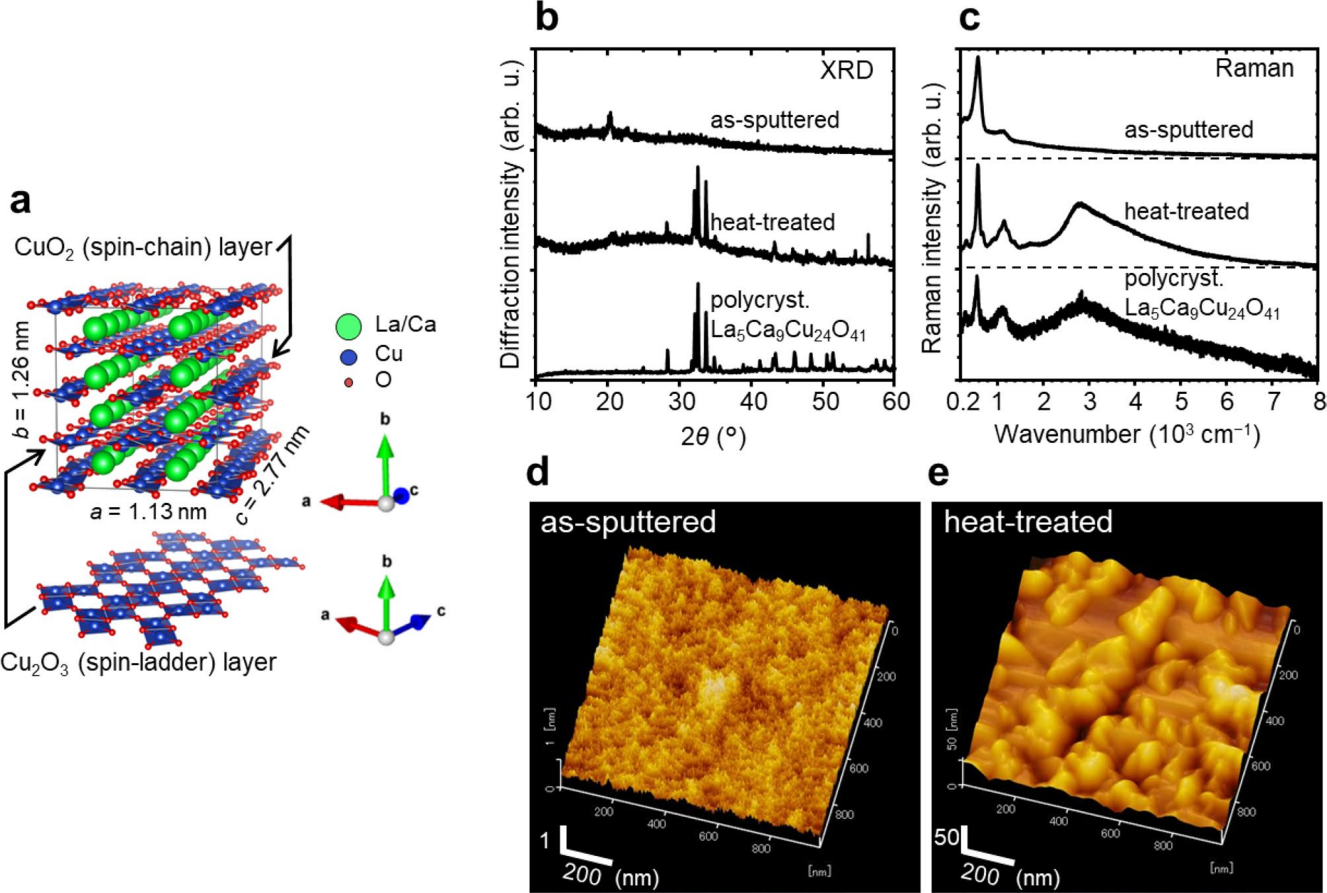
Structural characteristics of the La–Ca–Cu–O film. (**a**) A unit cell of the spin-chain ladder cuprate La_5_Ca_9_Cu_24_O_41_ and a Cu_2_O_3_ layer with spin ladders. The crystal structures are drawn using VESTA^27^ and referring to the structural data^28^. (**b**) XRD patterns and (**c**) Raman spectra of the as-sputtered and heat-treated (400 s at 700 °C) films and polycrystalline La_5_Ca_9_Cu_24_O_41_ synthesized by a solid-state reaction method^17^. (**d**,**e**) Surface morphology obtained by DFM of the as-sputtered and heat-treated films.

Spin-chain ladder cuprates such as La_5_Ca_9_Cu_24_O_41_ could be promising materials to achieve dynamic control of heat flow, since they possess unique thermal conduction due to magnons^7–10^ (for the structural property details, see the reviews by Hess^7^ and Vuletić et al.^11^). Briefly, La_5_Ca_9_Cu_24_O_41_, which possesses the highest magnon thermal conductivity at room temperature of these types of cuprates, consists of CuO_2_, La/Ca, and Cu_2_O_3_ layers stacked towards the *b*-axis (Fig. 1a); among them, the remarkable structure is the Cu_2_O_3_ layer composed of corner- and edge-sharing CuO_4_ squares (Fig. 1a), in which the *S* = 1/2 spins of Cu^2+^ form spin ladders^11^. In the ladders, magnons, corresponding to excitations of the singlet state of the paired electron spins to the triplet state, act as heat carriers similar to phonons and conduction electrons along the ladders, i.e., towards the *c*-axis in Fig. 1a, yielding high anisotropic thermal conductivity^7^. Notably, the magnon thermal conductivity is known to be sensitive to the hole density in the spin ladders^7^. For example, Sr_14_Cu_24_O_41_ and La_5_Ca_9_Cu_24_O_41_ have the same spin ladder structure (Fig. 1a), but their magnon thermal conductivity at room temperature, with values of ~ 10 and ~ 90 W/(m K)^7^, respectively, differ by nearly an order of magnitude. This difference is attributable to the difference in the hole density in the spin ladders; that is, while La_5_Ca_9_Cu_24_O_41_ has no holes in the spin ladders, Sr_14_Cu_24_O_41_ has self-doped holes (approximately one hole in the spin ladder per formula unit), leading to a decrease in the mean free path of magnons by magnon-hole scattering and resulting in a drastic decrease in the thermal conductivity^7,11^. These cuprates also possess phonon thermal conductivity, but the value slightly depends on the composition or transfer direction, which is limited to a few W/(m K)^7^.

The sensitivity to the hole density is a remarkable feature because dynamic control of the hole density could enable dynamic control of heat flow. Static control by chemical and defect engineering has been widely researched^7^; however, no research focusing on its dynamic controllability has been conducted yet. To demonstrate dynamic control, we prepared multi-layered samples that include an interface between an ionic liquid (IL) widely used for iontronics, *N*,*N*-diethyl-*N*-methyl-*N*-(2-methoxyethyl)ammonium bis(trifluoromethanesulfonic)imido (DEME-TFSI)^12–14^, and a spin-chain ladder system, a La–Ca–Cu–O (LCCO) polycrystalline film, fabricated by radio-frequency (rf) sputtering and post-annealing, where we initially expected that an electric double layer would yield a dense accumulation of holes^13^, resulting in a decrease in thermal conductivity. Clearly, epitaxial or highly oriented films^15–17^ and the single crystal usually grown by the travelling solvent floating zone method^8,18^ should be used to take advantage of their high, anisotropic thermal conduction, but we consider that the use of the polycrystalline film is preferable for increasing the active interfacial area, as described in the Discussion section, and important for practical applications^19,20^. The results showed that a voltage application causes imperfectly recoverable decreases in the thermal conductance of the film and the peak due to magnons in the Raman spectra, which were evaluated by in situ frequency-domain thermoreflectance (FDTR) and Raman spectroscopy under voltage application, respectively. This result can be interpreted by an increase in the hole density in the spin ladder. This report reveals that the dynamic control of magnon thermal conduction is a promising method for advanced thermal management applications.

## Results

### Structural investigation of La–Ca–Cu–O films

First, we show the fundamental structural properties of LCCO films. Figure 1b shows the X-ray diffraction (XRD) patterns of the as-sputtered and heat-treated (400 s at 700 °C) LCCO films with a thickness of ~ 500 nm deposited on the Si substrate. (See the “Methods” section regarding the sample preparation details). The as-sputtered film exhibits two small peaks at 2*θ* ~ 20.5° and 23.0°, which may be due to amorphous and/or nanocrystalline SiO_2_ (tridymite)^21^ formed on the substrate surface. A halo pattern seen in the range of 15–45° indicates that the LCCO film is in an amorphous and/or nanocrystalline state. On the other hand, the heat-treated film shows a halo pattern and sharp peaks corresponding to a powder pattern for the La_5_Ca_9_Cu_24_O_41_ polycrystal^18^. The peaks due to SiO_2_ become insignificant after the heat treatment.

The Raman spectra of the LCCO films are shown in Fig. 1c. For the as-sputtered film, we see three peaks at ~ 300, ~ 580 and ~ 1,100 cm^−1^. After the heat treatment, all the peaks become sharp, and a remarkable broad peak centred at ~ 2,900 cm^−1^ appears. The overall spectrum shows good agreement with that of the powdered La_5_Ca_9_Cu_24_O_41_ polycrystal. Considering that the penetration depth of the probe light with a wavelength of 532 nm for the LCCO film is ~ 130 nm (Supplementary Fig. S1), these results suggest that the amorphous and/or nanocrystalline LCCO has been crystallized by heat treatment from the surface through a deeper area than the penetration depth, which is consistent with the XRD results (Fig. 1b). Here, the peaks at ~ 300 and ~ 580 cm^−1^ can be assigned to A_g_ modes related to Cu and O, respectively^22,23^, in the Cu_2_O_3_ and CuO_2_ layers, and the peak at ~ 1,100 cm^−1^ is due to two-phonon modes^22^. The broad peak centred at 2,900 cm^−1^, referred to as the two-magnon peak, is unique to materials having antiferromagnetically coupled spins such as La_5_Ca_9_Cu_24_O_41_^24–26^, and its appearance suggests the presence of magnons. Figure 1 also shows the surface morphology observed by dynamic force microscopy (DFM). The as-sputtered film has a smooth surface (Fig. 1d). After the heat treatment, we see plate-like domains with a size of ~ 100 nm, which can correspond to randomly oriented La_5_Ca_9_Cu_24_O_41_ crystals (Fig. 1e).

### In situ Raman spectroscopy under voltage application

**Figure 2:**
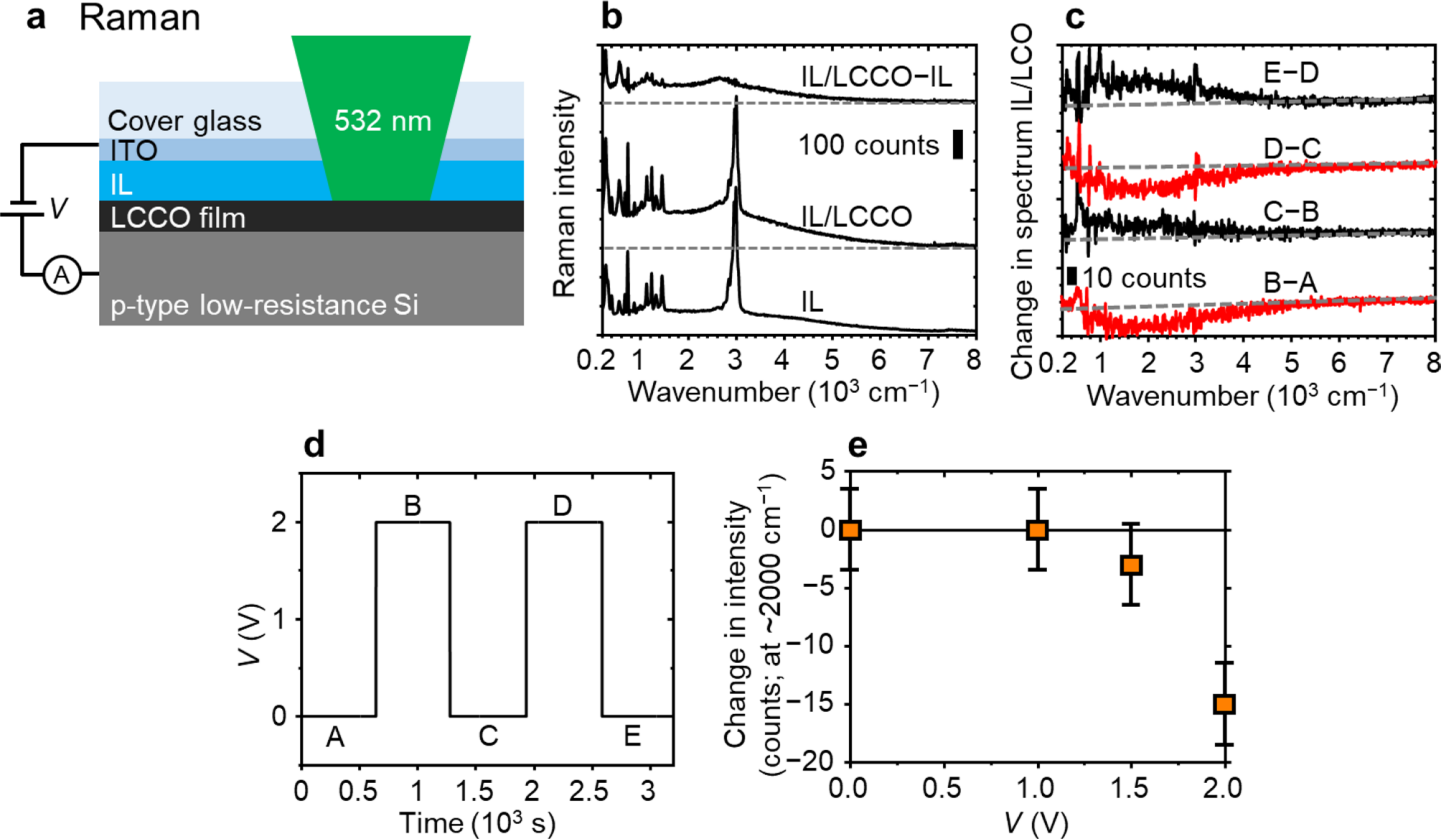
In situ Raman spectroscopy under voltage application. (**a**) Schematic cross section of the sample, which is drawn neglecting the light refraction. (**b**) Raman spectra when *V* = 0 V and when the laser spot is focused on the half-depth area of the ionic liquid (IL spectrum) and the interface between the ionic liquid and the LCCO film (IL/LCCO spectrum). The IL/LCCO–IL spectrum shows an increase in intensity compared with the IL and IL/LCCO spectra, where IL/LCCO has been normalized so that the peak at ~ 3,000 cm^−1^ in DEME^+^^30^ disappears in the IL/LCCO–IL spectrum. (**c**) Change in the IL/LCCO–IL spectrum by repetitive voltage application and short-circuiting, as shown in (**d**). (**e**) *V* dependence of the change in intensity at ~ 2,000 cm^−1^.

Figure 2a depicts a schematic cross section of the sample to measure changes in the Raman spectra of the LCCO film by voltage application (see the “Methods” section for the sample preparation). First, we measured spectra by focusing the laser spot on the half-depth area of the ionic liquid and the interface between the ionic liquid and the LCCO film using the setup with *V* = 0 (denoted as IL and IL/LCCO in Fig. 2b, respectively). For both spectra, sharp peaks originated from TFSI^−^ (< 2,000 cm^−1^) and DEME^+^ (~ 3,000 cm^−1^; C-H stretching)^14,29,30^. The spectrum denoted as IL/LCCO − IL shows an increase in intensity compared with the IL and IL/LCCO spectra, where the IL/LCCO spectrum has been normalized so that the peak at ~ 3,000 cm^−1^ due to DEME^+^^30^ disappears in the IL/LCCO − IL spectrum. In this spectrum, we clearly see the three peaks at ~ 580, ~ 1,100, and ~ 2,900 cm^−1^, corresponding to those of the heat-treated LCCO film (Fig. 1c), which means that the IL/LCCO spectrum includes information on the LCCO film. (However, the peaks due to TFSI^−^ remain at a wavenumber of < 2,000 cm^−1^, which is mentioned in the “Discussion” section.) Therefore, we examined changes in the IL/LCCO spectrum (Fig. 2c) by voltage application (Fig. 2d). In Fig. 2c, we see that the broad band centred at ~ 2,000 cm^−1^ is reduced by ~ 20% under a voltage application of *V* = 2 V with respect to the intensity at ~ 2,000 cm^−1^ in the IL/LCCO − IL spectrum and is recovered by short-circuiting. For the second voltage application and its release (spectra D–C and E–D, respectively), we see the appearance of sharp peaks due to DEME^+^ and TFSI^−^. Considering that the reduced broad band centred at ~ 2,000 cm^−1^ is included in the two-magnon peak region and that the two-magnon peak can be reduced by the presence of holes in the spin ladders^24,26^, these results suggest that the hole density in the spin ladders is increased by an applied voltage and recovered by short-circuiting. Interestingly, the peak position of the reduced band (~ 2,000 cm^−1^) was shifted to a lower wavenumber than the original one of the two-magnon peak (~ 2,900 cm^−1^). This shift may be explained by the presence of two types of magnons and selective disappearance by the voltage application: According to a theoretical analysis for spin-ladder cuprates^31^, spectral density of two-magnon state depend on the coupling direction of spins, i.e., along rungs and legs in the spin ladders, and the coupling along rungs yields a spectrum wide-based to a lower wavenumber compared to one along legs^31^. On the basis of this analysis, we consider that the holes in the spin ladders preferentially reduce the spectral density of two-magnon state derived from the coupling along rungs, which may be caused by magnon-hole coupling as is suggested by Sugai and Suzuki^24^, leading to the ununiform decrease of the two-magnon peak. As shown in Fig. 2e, the change in intensity at ~ 2,000 cm^−1^ is remarkably reduced when *V* exceeds ~ 1.5 V.

### In situ FDTR under voltage application

**Figure 3:**
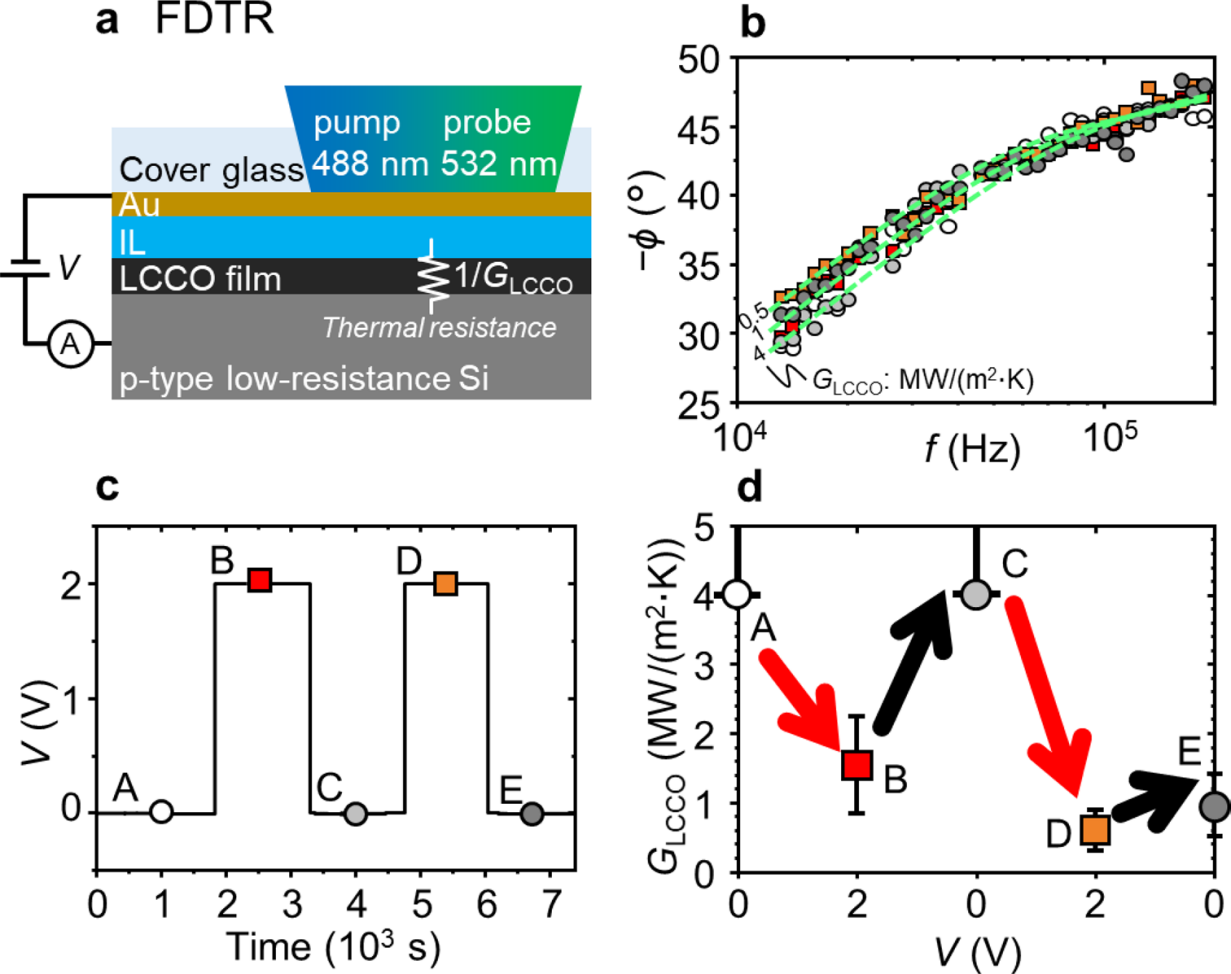
In situ FDTR under voltage application. (**a**) Schematic cross section of the sample, which is drawn neglecting the light refraction. 1/*G*_LCCO_ is the thermal resistance between the ionic liquid and the Si substrate; (**b**) *f* dependences of − *ϕ* and its response to voltage application shown in (**c**), where the symbols correspond to those in (**c**). In (**b**), the dashed lines are the simulated curves of the analytical solution for *G*_LCCO_ = 0.5, 1, and 4 MW/(m^2^ K). (**d**) Change in *G*_LCCO_ by voltage application. The circles for A and C denote the lower limits of *G*_LCCO_.

We performed FDTR measurements under voltage application using the setup in Fig. 3a (see the “Methods” section for the measurement and analysis details). Figure 3b shows the phase lag − *ϕ* of the probe light with respect to the pump light as a function of the modulation frequency *f* of the pump light and its response to voltage application (Fig. 3c), where the symbols correspond to those in Fig. 3c. The response to the voltage application seems negligible at *f* ~ 2 × 10^5^ Hz and increases with decreasing *f*. For the analytical fitting of the phase lag data for A (the initial state), four undetermined parameters exist: the interfacial thermal conductance between the cover glass and the Au film (*G*_g/Au_) and that between the Au film and the ionic liquid (*G*_Au/IL_), the thickness of the ionic liquid (*d*_IL_), and thermal conductance between the ionic liquid and the Si substrate (*G*_LCCO_) (the other parameter settings for the fitting are listed in Supplementary Table S1). The undetermined parameters were determined as follows. First, *G*_g/Au_ was determined to be ~ 50 MW/(m^2^ K) using data at *f* > 10^5^ Hz, where the value seems plausible, referring to a previous report^32^. Then, *G*_Au/IL_ was fixed at 50 MW/(m^2^ K) because *G*_Au/IL_ is presumed to be > 10 MW/(m^2^·K)^33,34^, and we found that the contribution of *G*_Au/IL_ to the phase lag at *f* ~ 10^4^–10^5^ Hz is negligible when *G*_Au/IL_ > 10 MW/(m^2^ K). Finally, *d*_IL_ and *G*_LCCO_ for A were determined to be ~ 700 nm and $\gtrsim$ 4 MW/(m^2^ K), respectively, by analytical fitting. Under this condition, the phase lag is sensitive to a *G*_LCCO_ value less than ~ 4 MW/(m^2^ K) in the *f* range of 10^4^–10^5^ Hz, as shown by the dashed lines in Fig. 3b. This sensitivity is reasonable because the penetration depth *d*_p_ of the temperature wave for the *f* range is estimated as 0.5–1.6 μm, using the thermal diffusivity *D* of the ionic liquid of ~ 8 × 10^−8^ m^2^/s^35^ and the equation ${d}_{\mathrm{p}}=\sqrt{\frac{D}{\uppi f}}$^36^, and the depth range includes the range between the ionic liquid and the Si substrate*.*

This fact enables estimation of *G*_LCCO_ by the analytical fitting of B–E in Fig. 3b. Figure 3d shows the change in *G*_LCCO_ by repetitive voltage application and short-circuiting (Fig. 3c). The initial application (A) causes a decrease by at least 40%, which is recovered by the subsequent short-circuiting (B). The subsequent voltage application (C) gives rise to a decrease of one order of magnitude in *G* (D) that seems to be imperfectly recovered (E).

## Discussion

Using the LCCO polycrystalline film and an ionic liquid, we demonstrated that the two-magnon peak in Raman spectra, supporting the presence of magnons, which are the major heat carriers, and the thermal conductance of the film can be dynamically controlled by voltage application (Figs. 2c, 3d). To explain these phenomena, we propose a tentative structural and response model as follows.

**Figure 4:**
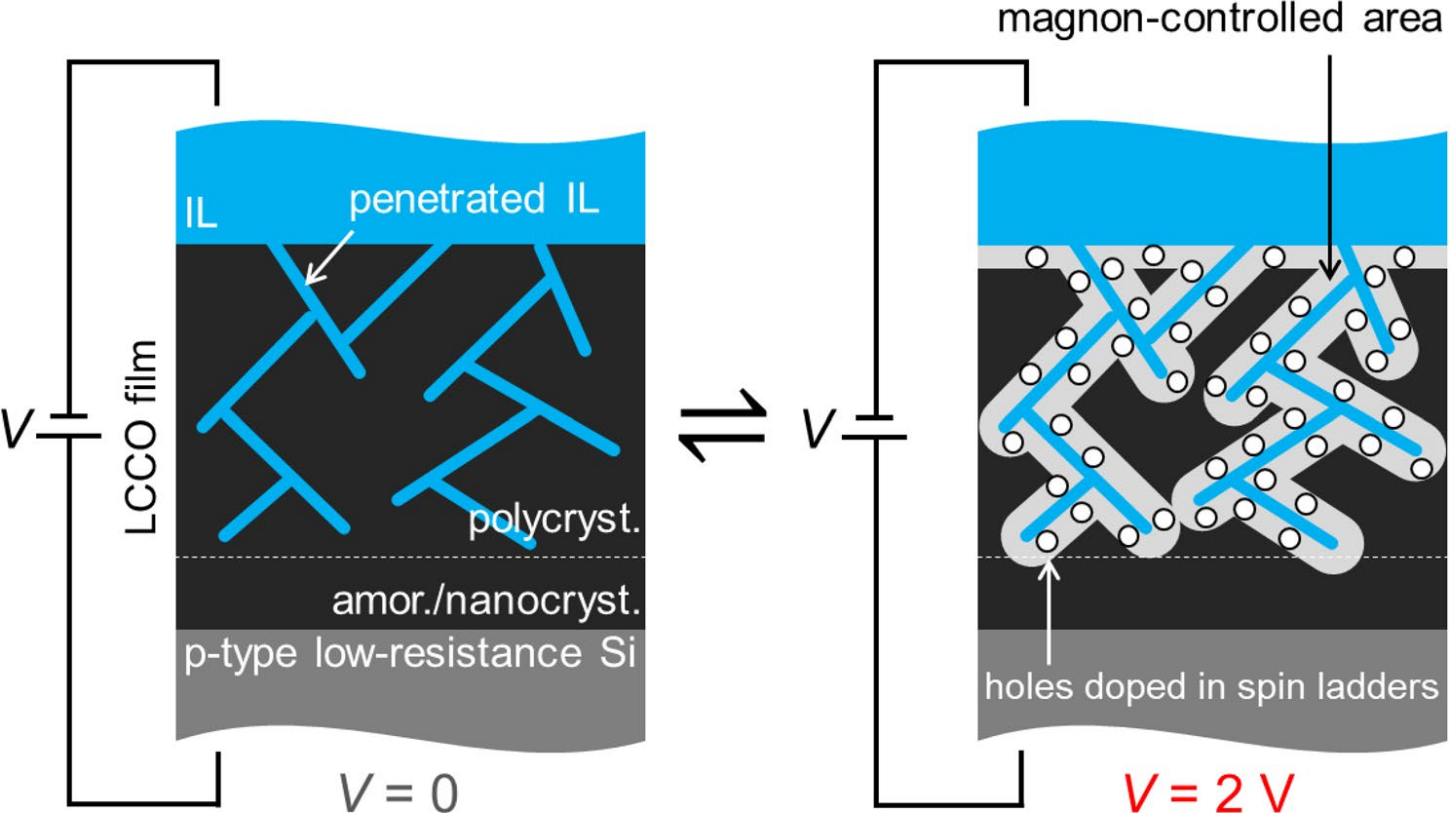
Schematic cross section of the LCCO film with *V* = 0 and *2* V.

Figure 4 (*V* = 0) shows a schematic cross section of the sample when *V* = 0 focused on the LCCO film, in which we infer that (a) the amorphous and/or nanocrystalline LCCO layer with a thickness of ~ 100 nm remains on the Si substrate, (b) cracks formed through the domain boundaries exist in the polycrystalline LCCO layer before application of the ionic liquid, and (c) TFSI^−^ in the applied ionic liquid preferentially penetrates into the polycrystalline layer through the cracks, meaning that a complicated series–parallel combination heat circuit consisted of the LCCO domains and the ionic liquid is formed. For (a), the XRD (Fig. 1b) and transmission electron microscopy (TEM) (Supplementary Fig. S2) results support the presence of the layer. For (b), the randomly oriented crystallization (Fig. 1b) of La_5_Ca_9_Cu_24_O_41_ with an anisotropic structure (Fig. 1a) can cause volumetric strain, resulting in cracks. The cleavage at the surface seen in Fig. 2e may support this assumption. Finally, (c) is deduced from the Raman spectroscopy result, i.e., the peaks due to TFSI^−^ remain in the LCCO/IL–IL spectrum, whose origin is unclear, but the p-type conductivity of La_5_Ca_9_Cu_24_O_41_ may be involved.

The recoverable dynamic changes by voltage application can be qualitatively interpreted as follows: Considering the transient current during the short-circuiting process after the voltage application to the sample (Supplementary Fig. S3) and referring to the capacitance of the DEME-TFSI/semiconductor interface^12–14,37^, the charged hole density *n*_A_ is estimated to be 10^13^–10^14^ cm^−2^ at most. Here, we need a hole density of approximately one hole in the spin ladder per formula unit at least to change the two-magnon peak and the magnon thermal conductivity of La_5_Ca_9_Cu_24_O_41_, as described in the Introduction section, so that the volumetric number density *n*_V_ is obtained as ~ 1 × 10^21^ cm^−3^ considering that the unit cell (Fig. 1a) consists of four formula units^28^. Thus, the fractional volume with the reduced magnon thermal conductivity, (*n*_A_/*n*_V_)/*d*_LCCO_, is evaluated to be ~ 0.2%, where *d*_LCCO_ is the thickness of the polycrystalline layer. However, this value seems significantly small to change the two-magnon peak by ~ 20% (Fig. 2c) and the thermal conductance of the LCCO film by ~ 40% (Fig. 3d). Therefore, there is a possibility that different factors affect the magnons in the whole film. One of the possible factors is the presence of holes hidden in the CuO_2_ (spin-chain) layer (Fig. 1a). That is, La_5_Ca_9_Cu_24_O_41_ also possesses holes in the CuO_2_ layers per formula unit (corresponding to ~ 1 × 10^21^ cm^−3^) but not in the Cu_2_O_3_ (spin-ladder) layer^11^. In the ground state, the holes in the CuO_2_ layers do not disturb the magnons, but under the voltage application, we assume that they are excited to the spin ladders by the electrostatic force with the preferentially penetrated TFSI^−^. The threshold voltage of ~ 1.5 V in Fig. 2e may support this assumption, because this value nearly corresponds to a difference in Madelung energy between oxygens of the Cu_2_O_3_ layers and the CuO_2_ layers, 1–2 eV, where the oxygens of the CuO_2_ layer has the lower energy, leading to the preferable presence in the CuO_2_ layers of the holes in the ground state^38^. If the ~ 10-nm thick areas of the LCCO domains with a size of ~ 100 nm surrounded by the ionic liquid are controllable, the volume fraction of the controlled area is estimated to be ~ 30%, which is comparable to the changes in the two-magnon peak and the thermal conductance, although it is difficult to validate the thickness of ~ 10 nm. The significant decrease in *G*_LCCO_ by the second application of voltage and its imperfect recovery (D and E in Fig. 3d, respectively) may be related to an overpenetration of the ionic liquid with the low thermal conductivity, as indicated in Fig. 2c, and a resultant irreversible structural change. The origins of the complicated spectral change at < 1,000 cm^−1^ in Fig. 2c have been unclear, but irreversible changes in the quantity of penetrated TFSI^−^ and the phonon mode by hole doping^39^ may be dominant factors.

In summary, we demonstrate the electric control of the two-magnon peak and the thermal conductance of the LCCO film using the ionic liquid. These change amounts were found to be significantly higher than expected from the density of the charged holes. Then, we propose a model in which the ionic liquid penetrates through the cracks formed through the domain boundaries, and the magnons are dynamically controlled throughout the film, which may be due to the excitation of holes in the CuO_2_ (spin-chain) layers to the Cu_2_O_3_ (spin-ladder) layers. We have presented no evidence of the magnon transport, and the results of the Raman spectroscopy just suggest the presence of the magnons and its change, not including information on changes in their mean free path or anisotropy. To elucidate it, we need to investigate the dynamic control using single crystals and measure their life time. Although obtaining a quantitative understanding and achieving high reversibility will be future work, this report reveals that spin-chain ladder cuprates have potential for the development of advanced thermal management applications.

## Methods

### Sample preparation and fundamental characterization

The LCCO films were deposited on p-type low-resistance (001) Si substrates working as a bottom electrode by using a conventional rf magnetron sputtering system (EB1100, Canon Anelva). The deposition was carried out for 50 min in pure Ar gas with a pressure of 0.5 Pa without substrate heating. The sputtering target was sintered polycrystalline La_5_Ca_9_Cu_24_O_41_ (Toshima Manufacturing Co., Ltd.) that was synthesized by a solid-state reaction method following a previous report^17^. The deposited film was heat-treated at 700 °C for 400 s for crystallization in ambient air by using an electric furnace with a heating rate of 10 °C/min and with slow cooling in the furnace. The heat-treated film was confirmed to be ~ 500-nm-thick and a p-type semiconductor by means of the Hall effect measurement. The thicknesses of all the films except for the ionic liquid and the surface morphology of the LCCO films were obtained by DFM (Nanocute, Hitachi High-Tech Corporation). For the LCCO films before and after heat treatment, the XRD patterns were recorded with a New D8 Advance apparatus (Bruker) using Cu*K*_α_ radiation and a *θ*-2*θ* configuration. Polycrystalline La_5_Ca_9_Cu_24_O_41_ used as a reference in XRD and Raman spectroscopy was synthesized in the same way as the sputtering target.

For in situ Raman spectroscopy (Fig. 2a), an ionic liquid, DEME-TFSI (Kanto Chemical Co., Inc.), was dropped onto the heat-treated film with 150-μm-thick spacers (polyimide tape) and subsequently covered by a cover glass with a 200-nm-thick ITO film deposited on the bottom surface by rf sputtering. For in situ FDTR, the ionic liquid was coated on the heat-treated film, followed by placing a cover glass with a 100-nm-thick Au film deposited on the bottom surface by DC sputtering. The ITO and Au films work as top electrodes and at the same time as a transparent window for Raman spectroscopy and a transducer for FDTR, respectively. These top electrodes and the conductive Si substrate were connected to a source meter (2450, Keighley) using thermosetting Ag paste and electrical wires, and the voltage and current were recorded. The active electrode area for both measurements was ~ 1.0 cm^2^.

### Raman spectroscopy

Raman spectra were obtained using a conventional system (NRS-4500, JASCO) at room temperature, including confocal microscopy with a 20 × objective lens and an excitation source of a 532-nm laser light. The scattered light was detected with a backscattering configuration with no polarizer, while the light incident to the samples was linearly polarized. For the in situ observation under the voltage application, the intensity was recorded for 300 s after the transient current became nearly stable, which was 200–300 s after voltage switching (see Supplementary Fig. S3), and the same is true for in situ FDTR.

### FDTR

FDTR measurements were performed at room temperature with reference to a setup by Regner et al*.* (Fig. 1a in Ref.^40^). In our study, pump and probe lights with wavelengths of 488 and 532 nm were emitted from a laser-diode module (PhonX+, Omicron) and a diode-pumped solid-state laser module (GLK 32200 TS, LASOS), respectively, in which the pump light was directly sinusoidally modulated with a frequency of *f* by a function generator (33612A, Keysight Technologies). The phase lag with respect to the pump light, − *ϕ*, of the probe light modulated by the sinusoidal heating was detected by using a dual phase lock-in amplifier (LI5660, NF Corporation). The parameters used for the analysis are listed in Supplementary Table S1, in which the thermal conductivity of the Au film was evaluated with the Wiedemann–Franz law^41^ and the laser beam radius was measured by the knife-edge method^42^.

Generally, the phase lag data are fitted via nonlinear least-square algorithms to the analytical solution of the heat equation for the multi-layered structure whose top surface is sinusoidally heated^36,43^, which we basically followed in this work. However, in our case (Fig. 3a), the transducer, the Au film, is placed under the cover glass, and accordingly, the heat dissipation towards the cover glass is not negligible. Therefore, we modified the heat equation model as follows: first, we separate the total heat flux *f*_t_ inflowing to the top surface of the Au film into that towards the cover glass and towards the Si substrate, *f*_d_ and *f*_u_, where the subscripts d and u denote downward and upward, respectively, meaning that *f*_t_ = *f*_d_ + *f*_u_. From the same insight as the traditional model^36,43^, we obtain the relation *θ*_t_ = (− *D*_d_/*C*_d_) *f*_d_ = (− *D*_u_/*C*_u_) *f*_u_, where *C*_u_ and *D*_u_ correspond to the elements of the multiplied matrix in Ref.^36^ and are related to the interfacial thermal conductance between the Au film and the cover glass and the thermal conductivity, specific heat, and thickness of the cover glass. These relations yield *θ*_t_ = − *f*_t_/[*C*_d_/*D*_d_ + *C*_u_/*D*_u_], corresponding to the replacement of *C*/*D* in the traditional model with *C*_d_/*D*_d_ + *C*_u_/*D*_u_.

## Supplementary information

Supplementary Information

## Data Availability

The authors declare that the data supporting the findings of this study are available within the article.
