# Supplementary material for: Dynamic control of heat flow using a spin-chain ladder cuprate film and an ionic liquid
Source: Sci Rep. 2020 Sep 2;10:14468. doi: 10.1038/s41598-020-70835-z (PMC7468102; doi:10.1038/s41598-020-70835-z)
Supplement: Supplementary file 1 — Supplementary Information [file 41598_2020_70835_MOESM1_ESM.docx]

**Supplementary information for the manuscript entitled “Dynamic control of heat flow using a spin-chain ladder cuprate film and an ionic liquid”**

Nobuaki Terakado^1,2^,* Yoshinori Nara^1^, Yuki Machida^1^, Yoshihiro Takahashi^1^, Takumi Fujiwara^1^ *

*^1^Department of Applied Physics, Tohoku University, 6-6-05 Aoba, Aoba-ku, Sendai 980-8579, Japan*

*^2^PRESTO, Japan Science and Technology Agency, 4-1-8 Honcho, Kawaguchi 332-0012, Japan*

*Corresponding and requests for materials should be addressed to N.T. (email: terakado@laser.apph.tohoku.ac.jp) or T.F. (email: fujiwara@laser.apph.tohoku.ac.jp)


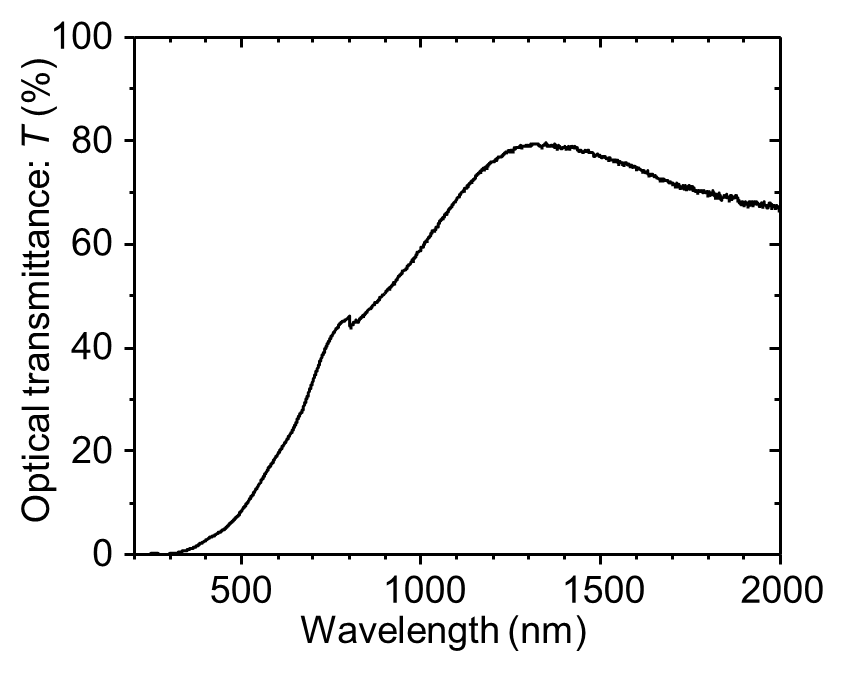


**Supplementary Fig. 1.** Optical transmission spectrum of a heat-treated (400 s at 700 °C) La–Ca–Cu–O (LCCO) film with a thickness *d* of ~250 nm deposited on a SiO­_2_ glass substrate obtained by UV-Vis-NIR spectrophotometry. The dip at the wavelength of 800 nm is from the instrument. The optical transmittance *T* is approximated by *T* = *T*_0_exp(−*αd*), where *α* is the absorption coefficient of the LCCO film and *T*_0_ is the transmittance for *α* = 0, which is assumed to be ~80% from the transmittance at the wavelength of ~1300 nm. For the 532-nm probe light in Raman spectroscopy, *T* is 12%, and accordingly, the penetration depth *α*^−1^ is obtained as ~130 nm.

**Supplementary Table 1**. Parameters set for the analytical fitting in frequency-domain thermoreflectance (FDTR). (**upper table**) Wavelength and beam radius of the pump and probe light. (**lower table**) Thickness *d*, thermal conductivity *K*, volumetric specific heat *C* of each layer, and thermal conductance *G*_LCCO_, where the layers and interfaces are listed in order from the top to the bottom. *G*_g/Au_, *G*_Au/IL_, *d*_IL_, and *G*_LCCO_ are the initial undetermined parameters.

|  | *λ* (nm) | *w* (μm) |
| --- | --- | --- |
| Pump | 488 | 18* |
| Probe | 532 | 23* |

|  | *d* | *K* (W/(m K)) | *C* (MJ/(m^3^ K)) | *G* (MW/(m^2^ K)) |
| --- | --- | --- | --- | --- |
| Cover glass | 150 μm | 1.0^1^ | 2.1^1^ | – |
| Interface | – | – | – | *G*_g/Au_ = 50 |
| Au | 80 nm | 220* | 2.5^1^ | – |
| Interface | – | – | – | *G*_Au/IL_ = 50 |
| IL (DEME-TFSI) | *d*_IL_ = 700 nm | 0.13^2^ | 1.9^2^ | – |
| LCCO film | 500 nm | – | – | *G*_LCCO_ $\gtrsim$ 4** |
| Si | 500 μm | 150^1^ | 1.6^1^ | – |

* See Methods in the main text regarding the evaluation.

** For the initial state A; see Fig. 3 in the main text.


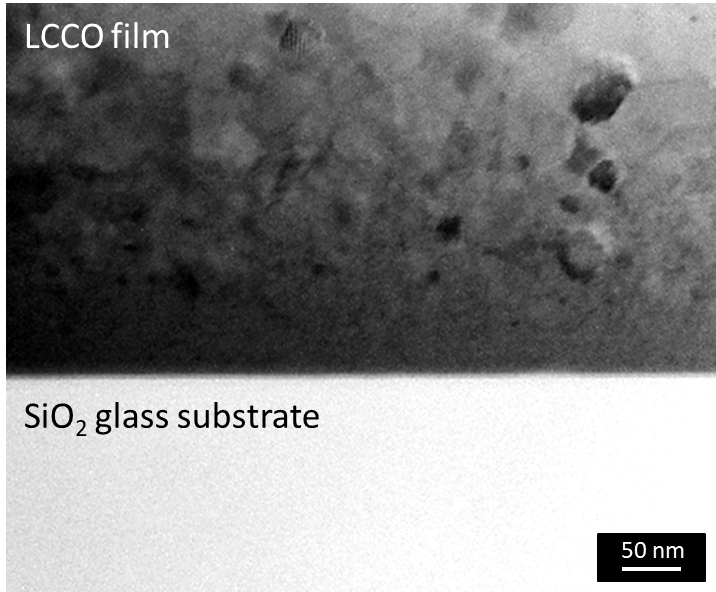


**Supplementary Fig. 2.** Cross-sectional transmission electron microscopy image near the substrate surface of an LCCO film sputtered on a SiO_2_ glass substrate. The sputtering conditions are the same as those in the main text, except for the substrates. A homogeneous area with a thickness of ~100 nm on the substrate is observed, which is expected to be amorphous/nanocrystalline LCCO, and the subsequent deposition of the polycrystals whose size increases as the depth becomes shallower.


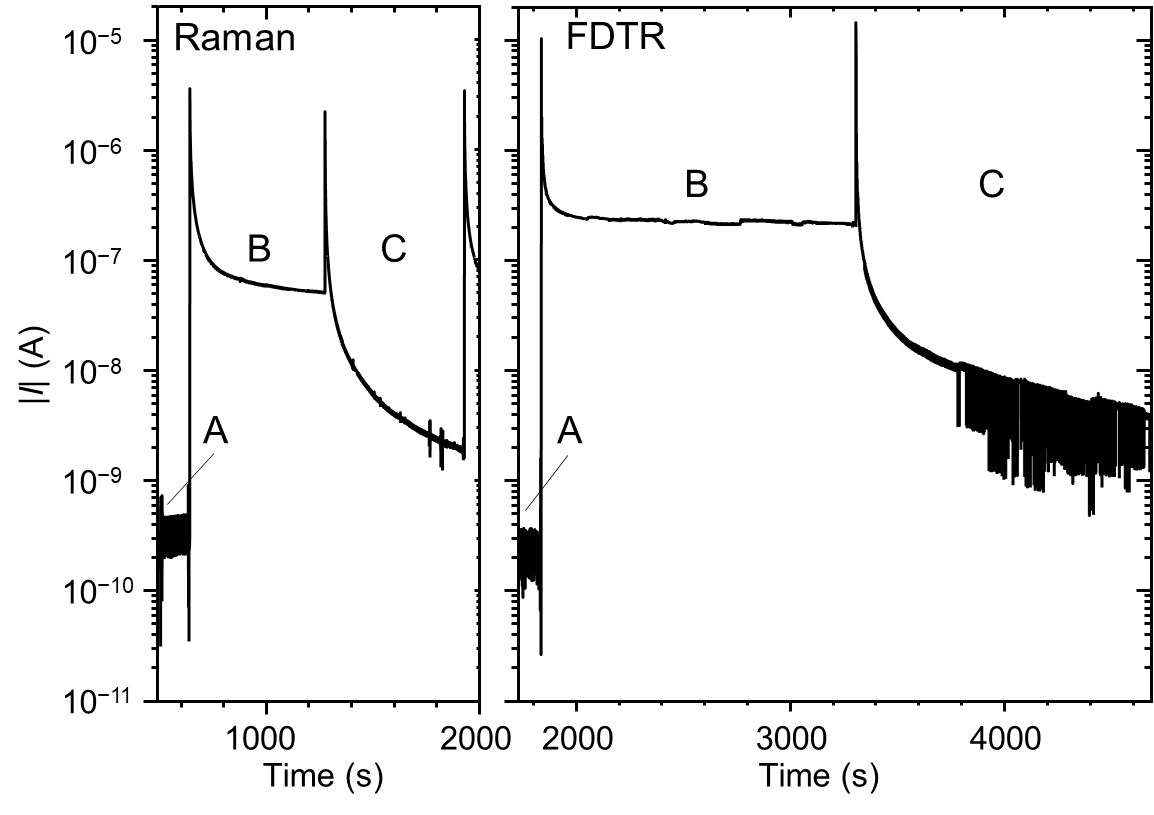


**Supplementary Fig. 3.** Time dependence of the charging and discharging current ­|*I|* for the voltage application, corresponding to Fig. 2c (Raman) and Fig. 3d (FDTR) in the main text. For both measurements, the charged carriers are estimated to be ~10^14^ cm^−2^ from the transient current after switching voltages, and the leak current density is approximately 100 nA/cm^2^.

**References**

1. *CRC Handbook of Chemistry and Physics*. (CRC Press, 2016).

2. López-Bueno, C., Bugallo, D., Leborán, V. & Rivadulla, F. Sub-μL measurements of the thermal conductivity and heat capacity of liquids. *Phys. Chem. Chem. Phys.* **20**, 7277–7281 (2018).
